# Once-weekly administration of insulin in the real-world management of type 2 diabetes. A Delphi-like consensus

**DOI:** 10.1007/s00592-025-02619-8

**Published:** 2025-12-03

**Authors:** Riccardo Candido, Raffaella Buzzetti, Agostino Consoli, Concetta Irace, Enrico Torre, Roberto Trevisan, Gian Paolo Fadini, T2D once-Weekly Insulin Expert Panel Group, Cesare Berra, Paolo Di Bartolo, Katherine Esposito, Andrea Giaccari, Francesco Giorgino, Edoardo Mannucci, Salvatore Oleandri, Gianluca Perseghin, Giuseppina Russo, Sebastiano Bruno Solerte

**Affiliations:** 1 Department of Medical, Surgical and Health Sciences, University of Trieste, SC Patologie Diabetiche, Azienda Sanitaria Universitaria Giuliano Isontina, Trieste, Italy; 2 Department of Experimental Medicine, Sapienza University of Rome, Rome, Italy; 3 Department of Medicine and Ageing Sciences and CAST, University G. d’Annunzio di Chieti-Pescara, Chieti , Italy; 4 Department of Health Science, University Magna Grᴂcia Catanzaro, Catanzaro, Italy; 5 Diabetes and Metabolic Diseases Unit, ASL3 Genoa, Genoa, Italy; 6 Department of Medicine and Surgery, University of Milano Bicocca, Milan, Italy; 7 Department of Medicine, University of Padova, Padua, Italy; 8 Endocrinology and Diabetes Unit, ASST Papa Giovanni XXIII, Bergamo, Italy

**Keywords:** Type 2 diabetes, Once weekly insulin, Degludec, Glargine, Icodec, Disease management.

## Abstract

**Background:**

Despite major advancements in diabetes management, insulin therapy continues to have a prominent role in glycemic control, aiding numerous patients. However, treatment-associated unmet needs pose a hindrance to therapy acceptance and adherence, negatively affecting patient outcomes due to less effective glycemic management.

**Method:**

A consensus study was conducted using a Delphi-like methodology, with the aim of highlighting and discussing the potential benefits and challenges with the introduction of once-weekly basal insulin icodec in the management of diabetes.

**Results:**

The consensus firmly highlights the transformative approach and the timely adoption of once-weekly basal insulin for patients affected by type 2 diabetes. Once-weekly insulin icodec was broadly supported as a viable alternative to daily basal insulin, particularly for insulin-naïve individuals and those on basal-only regimens. Key advantages included reduced injection burden, improved adherence, and potential cost savings. The therapy was also seen as a way to counteract therapeutic inertia and improve quality of life. Although some implementation challenges were noted, namely patient selection and titration, most experts endorsed educational efforts and digital tools to support adoption. The panel supported the progressive replacement of daily with weekly basal insulin.

**Conclusion:**

The advent of once-weekly insulin icodec therapy is an unprecedent breakthrough in diabetes care. Compared with once-daily insulin analogues, it offers a simplified, secure, enhanced, and sustained glycemic control, counteracting therapeutic inertia, expectedly improving adherence to insulin therapy. Insulin icodec can not only enable personalized treatment and positively impact the clinical outcome, but also improve patient satisfaction and overall quality of life.

## Introduction

Diabetes is a major health issue with an increasing prevalence worldwide, posing challenges at different levels [1]. Diabetes imposes a burden on both the affected individuals and the health care systems [2], as it is associated with macrovascular (i.e., coronary heart disease, stroke and peripheral vascular disease) and microvascular (i.e., end-stage renal disease, retinopathy and neuropathy) complications [3]. To date, the number of people affected by diabetes globally has surpassed 800 million, calling for urgent actions to address the rising disease rates and treatment gaps [4].

While insulin therapy is vital for type 1 diabetes (T1D), in type 2 diabetes (T2D) it maintains a crucial role in achieving adequate glycemic control [5] and prevent disease progression [6]. Importantly, besides glycemic control, early initiation of insulin therapy in T2D has been shown to have beneficial outcomes in terms of reduced glucotoxicity, thereby improving insulin resistance and beta-cell function [7]. International and Italian national guideline for diabetes recommend insulin treatment when therapeutic goals are not met using other pharmacological strategies or when hyperglycemic symptoms and signs occur [8, 9]. However, insulin therapy is associated with a considerable burden, related to the daily injection and more so when multiple daily injections are required for effective treatment [10].

For decades, researchers have attempted to prolong the duration of basal insulin with the ultimate aim of mimicking endogenous insulin action through the engineering of biosynthetic molecules [11]. Although first-generation basal insulin analogues, namely insulin glargine (IGlar U100) and insulin detemir (IDet U100), exhibit prolonged exposure and less variability than NPH, they still require twice-daily dosing in some patients. This unmet need prompted the development of second-generation once-daily basal insulins such as IGlar U300 and degludec (IDeg U100 and IDeg U200) [11].

Considerable advancements have been made in the management of diabetes, including the availability of clinically effective once-daily basal insulin therapies, but challenges remain due to the complexity of treatment regimens requiring self-management and the negative impact on the quality of life of affected patients and caregivers. Insulin therapy is frequently delayed and started only when severe hyperglycemia occurs, and yields good glycemic control only in a low number of patients [12]. In fact, a meta-analysis demonstrated that only 42.8% of diabetes patients, globally, achieve optimal hemoglobin A1c (HbA1c) target [13]. Assessment of German real-world data, also, show that the time to initiation of insulin therapy increased in the primary care practice between 2010/2011 and 2016/2017, and that more than one third of patients received insulin only when A1c levels were above 9% [14]. Moreover, the time to insulin initiation has been reported to be at least 5 years, mainly due to fear of hypoglycemia upon insulin initiation or intensification, and it was associated with an A1c of 8.7–9.8% [15]. The same study summarized that insulin intensification has been linked with a reduced A1c by 1.4% and a decrease of hypoglycemic events. Indeed, the lower the A1c is at the start of basal insulin, the higher is the chance to achieve the A1c target [16], furthermore, starting insulin at a lower A1c is associated with less weight gain [17]. In a recent study, Giandalia A et al. evaluated temporal trends in insulin prescription and usage patterns over time, and reported a progressively earlier insulin treatment initiation among T2D patients during the observation period [18]. Nonetheless, the HbA1c levels were off-target for many patients after 12 months of insulin therapy.

Barriers limiting the initiation of insulin treatment may derive from concerns about potential adverse effects, such as hypoglycemia, weight gain, injection-related discomfort, need for glucose monitoring, and intensified disease management [19, 20]. A significant challenge in the management of diabetes is adherence and persistence to therapy, that can be undermined by the inconvenience of frequent injections, negatively affecting blood glucose control [10]. One third of patients do not adhere to their therapy [21], and, unfortunately, glycemic goals are achieved only in 5–45% of patients [11].

Ultimately, the need for a simplified treatment represents a further crucial requirement for individuals with diabetes. A recent research indicates that complex medication regimens in T2D are associated with poor adherence and less effective glycemic management [22]. Lower adherence, in turn, can lead to suboptimal glycemic control, resulting in an increased risk of complications, hospitalization, mortality, and health care costs [23].

To appropriately address these unmet needs, the development of third-generation basal insulins has been pursued, with the aim of retaining pharmacokinetic dynamics with an increased half-life, i.e., a longer duration of action, enabling once-weekly dosing and achieving efficient glycemic control [11]. Clinical data have demonstrated the efficacy and safety of once-weekly insulin icodec in the treatment of T2D patients, which has the potential to improve therapy adherence and persistence and alleviate treatment-associated burden while improving patients’ satisfaction and quality of life. A recent multi-stakeholder survey conducted by the Italian Diabetes Society, aimed at providing insight from the perspectives of patients, physicians and payers, concluded that the use of weekly basal insulin may reduce treatment burden and improve both adherence and quality of life, however, the implementation in the clinical practice requires further assessment of issues related to safety, efficacy and costs [24].

Two papers have recently focused on the economic evaluation of disease management and quality of life in people with diabetes using once weekly insulin icodec in the Italian National Health System (NHS), along with a budget impact analysis [25, 26]. Icodec resulted to improve the patient management and quality of life, without increasing the economic burden for the Italian NHS, and guaranteeing an excellent cost-effectiveness profile considering a five years budget impact.

The aim of this expert opinion paper is to provide an overview of the introduction of once-weekly administration of insulin in the management of T2D, and to discuss whether such basal insulin analogues can redefine the current insulin place in therapy.

## Methods

**Fig. 1:**
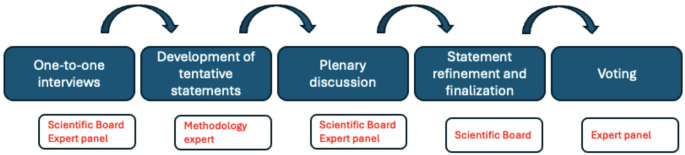
The roadmap of the Delphi-like consensus process

This study was conducted using a Delphi-like methodology [27, 28] by combining activities that limit verbal interactions with one-to-one meetings [29]. The aim of the consensus process was to gather insights and highlight the benefits of the once-weekly administration of basal insulin regimen in the treatment of T2D, as well as its potential impact on once-weekly insulin place in therapy. The Delphi-like process is summarized in Fig. 1.

Sixteen expert Italian diabetologists were invited to take part in this study, six of whom formed the scientific board, and the remaining 10 acted as the expert panel. The roles of the two groups were defined from the beginning, and their functions were harmonized so as to reduce possible bias: in the first phase, all of the experts underwent one-to-one interviews to generate expert opinions, with the aim of enhancing the collection of practical insights from the Italian clinical experience. Subsequently, based on the qualitative inputs gathered, a methodology expert (see acknowledgements section) developed a set of tentative statements that were subsequently discussed in a dedicated meeting including both the scientific board and the expert panel. Finally, the scientific board, based on the inputs received during the meeting, refined and finalized the statements before submitting them for formal voting via an online platform.

Tentative statements were developed according to content expressed by experts in the interviews, and clustered in the following topics:

- Role of second-generation basal insulin analogs in type 2 diabetes.
- Advantages of second-generation basal insulin analogs.
- Advantages of third generation once-weekly basal insulin in type 2 diabetes.
- Place in therapy of once-weekly basal insulin analogs.
- Elegibility in once-weekly basal insulin analog.
- Challenges for once-weekly basal insulin analogs.
- Future perspectives and technology.

The statements were rated, independently and anonymously, by the expert panel, using a five-point Likert scale as follows: 1, strongly disagree; 2, disagree; 3, neither agree nor disagree; 4, agree; and 5, strongly agree. A cut-off limit was set at 80%, i.e., consensus was defined as achieved agreement if at least 80% of the expert panel members rated a statement with a score of 4 or 5. Finally, the consensus results were analyzed and discussed by the advisory board.

## Results

All 10 panel members completed the online rating of all 38 statements independently and anonymously (100% response rate). The ≥80% agreement was achieved for 24 out of 38 statements. The statements and achieved consensus rates are presented in Table 1.

The achieved consensus confirmed that basal insulin remains essential in cases of severe glycemic decompensation (statement 2, 81.8% agreement) and plays a key role in preventing long-term complications for patients whose HbA1c levels remain above target despite optimized non-insulin therapies (statement 3, 100% agreement). Furthermore, when other medications fail to achieve optimal glycemic control, basal insulin remains a necessary therapeutic tool (statement 4, 100% agreement).

Second-generation basal insulins, such as degludec and glargine-300, provide superior glycemic control compared to first-generation basal insulin analogs (statement 5, 81.8% agreement). These newer formulations also reduce glycemic variability (statement 6, 100% agreement) and lower the risk of hypoglycemic events (statement 7, 100% agreement) compared to first-generation basal insulins. The ability of second-generation basal insulins to reduce the psychological burden of diabetes management, however, did not reach consensus (statement 8, agreement 54.5%), suggesting that while they may help, this effect is not strongly supported by all respondents, possibly because a certain degree of flexibility strikes with the daily administration. Similarly, the role of second-generation basal insulins in enhancing adherence due to greater flexibility in insulin titration was partially acknowledged but did not reach the consensus threshold (statement 9, 72.7% agreement). Only 40% (statement 10) voted that second-generation insulin favors early insulin treatment compared to first-generation insulin, which could be explained by the fact that current guidelines delay insulin initiation in favor of GLP-RA or sodium-glucose cotransporter 2 (SGLT-2) inhibitors despite the better safety profile of second-generation basal insulin. Another explanation may be that insulin treatment does not align with the respondents’ clinical practice or experience, or that insulin is not viewed as a possible early treatment by the physician.

Although not formally reaching 80% consensus (statement 12, 72.7% agreement), experts generally agreed that in people living with T2D, once-weekly basal insulin can replace daily basal insulin therapy, providing greater efficacy, particularly in insulin-naïve individuals or those on daily basal insulin therapy alone (i.e., without boluses). A major advantage of weekly insulin is the significant reduction in injection burden, from 365 injections per year to only 52, which is expected to improve adherence to therapy and facilitate patient acceptance (statement 14, 100% agreement).

Additionally, weekly insulin is believed to reduce therapeutic inertia, encouraging earlier initiation of insulin therapy (statement 15, 100% agreement), while also improving overall quality of life (statement 16, 100% agreement) and alleviating the workload for both caregivers and healthcare professionals (statement 17, 90.9% agreement).

From a clinical perspective, once-weekly basal insulin could help patients with suboptimal control on daily basal insulin achieve better glycemic levels with fewer injections (statement 21, 81.8% agreement). Combining once-weekly insulin with other weekly therapies could further simplify diabetes management (statement 23, 100% agreement). While clinical trials have demonstrated the efficacy of weekly basal insulin, real-world data will be necessary to confirm its definitive role in therapy, especially in diverse patient populations that can benefit from such treatment (statement 22, 90.9% agreement).

Economic considerations were also evaluated: while there may be a potential for cost savings in healthcare systems (statement 18, 63.3% agreement), this aspect did not reach consensus.

Furthermore, the introduction of once-weekly insulin therapy was recognized as a paradigm shift in diabetes treatment, providing a more flexible and patient-friendly approach (statement 19, 90.9% agreement). Experts also agreed that once-weekly insulin should be considered as an add-on therapy for patients not achieving glycemic targets with other treatments like metformin, GLP-1RAs, and SGLT-2i (statement 20, 90.9% agreement).

Patient selection was a key discussion point. Experts were against a statement that patients who are already on daily basal insulin and achieving good metabolic control should not be switched to once-weekly insulin treatment (statement 26, only 18.2% agreed, implying that 81.8% opposed this restriction). Experts did not agree that individuals struggling with insulin administration, such as elderly patients supported by caregivers or those with irregular lifestyles, are priority candidates for once-weekly insulin treatment since consensus was not achieved (statement 27, 63.6% agreement). The rejection of the statement expressing that people engaging in intense physical activity should *not* be treated with once-weekly insulin (statement 33, 0% agreement) suggests that physical activity was not considered as a contraindication to icodec use. Similarly, the lack of agreement on statements suggesting challenges with hospitalization and acute illness (statement 34, 9.1% agreement) and difficulties in managing hypoglycemia (statement 35, 0% agreement) indicate that these concerns are not perceived as major barriers to implementation of once-weekly insulin administration.

Implementation challenges were acknowledged by the expert panel members, as they agreed on the need for educational programs for people living with T2D to support the adoption of weekly basal insulin (statement 32, 90.9% agreement), but opinions were more divided on aspects like patient selection and monitoring (statement 29, 45.5% agreement). In support of this rating, patient with cognitive decline, patients with very high HbA1c (i.e., > 11%), or patients requiring daily evaluation of insulin need are not ideal candidates. Regarding the use of different titration algorithms, compared to second-generation basal insulins, 36.4% (statement 31) voted that these pose a challenge in the implementation of once-weekly basal insulin therapy. The use of dedicated digital tools and continuous glucose monitoring (CGM) to optimize weekly insulin therapy was supported by 81.8% (statement 37) and 63.6% (statement 36) of experts, respectively.

Finally, the majority of the experts believe that once-weekly basal insulin can benefit all T2D patients who require basal insulin (statement 24, 90.9% agreement), including those on daily basal insulin and insulin-naïve patients (100% agreement), and could progressively replace daily basal insulin therapy (statement 38, 81.8% agreement), reinforcing the idea that it may become the new standard of care in the future. In the meantime, it is necessary to raise awareness for effective transition to once-weekly basal insulin (statement 30, 81.8% agreement).

**Table 1:** Statements rated by the expert panel

Statement	% achieved consensus
Role of second-generation basal insulin analogs in type 2 diabetes	
1. People living with type 2 diabetes have an insulin secretion deficit.	90.9%
2. Basal insulin therapy in type 2 diabetes is necessary in cases of severe glycemic decompensation.	81.8%
**3**. Basal insulin is effective in preventing long-term complications of diabetes in people whose HbA1c levels remain above target desite optimized non-insulin therapies.	100%
4. Basal insulin remains a fundamental therapeutic tool when other medications fail to achieve good glycemic control.	100%
Advantages of second-generation basal insulin analogs	
5. Second-generation basal insulins (degludec and glargine U300) provide a more effective glycemic control compared to first-generation basal insulins.	81.8%
6. Second-generation basal insulins (degludec and glargine U300) reduce glycemic variability compared to first-generation basal insulins.	100%
7. Second-generation basal insulins (degludec and glargine U300) reduce hypoglycemic events compared to first-generation basal insulins.	100%
8. Second-generation basal insulins (degludec and glargine U300) reduce the psychological burden of diabetes management.	54.5%
9. Second-generation basal insulins (degludec and glargine U300) enhance adherence by greater flexibility in insulin titration compared to first-generation basal insulin.	72.7%
10. Second-generation basal insulins (degludec and glargine U300) favors the early insulin treatment compared to the first-generation basal insulin.	40%
11. Second-generation basal insulins (degludec and glargine U300), in combination with as-needed GLP-1 receptor agonists, provide better glycemic control compared to first-generation basal insulins. They also reduce weight gain and allow for lower insulin doses compared to use without combination.	81.8%
Advantages of third generation once-weekly basal insulin in type 2 diabetes	
12. In people living with type 2 diabetes, once-weekly basal insulin can replace daily basal insulin therapy, providing greater efficacy, particularly in insulin-naïve individuals or those on basal insulin therapy alone.	72.7%
13. In people living with type 2 diabetes, once-weekly basal insulin can replace daily basal therapy, making disease management less burdensome.	100%
14. The lower injection frequency (52 vs. 365 injections per year) will improve adherence and facilitate acceptance among people living with type 2 diabetes.	100%
15. Once-weekly basal insulin may contribute to promote earlier initiation of basal insulin therapy thus reducing therapeutic inertia.	100%
16. Once-weekly basal insulin reduces the impact of therapy on patients’ quality of life.	100%
17. Once-weekly basal insulin could alleviate the workload for caregivers and healthcare professionals.	90.9%
18. The use of once-weekly basal insulin will lead to cost savings for the national healthcare system.	63.3%
19. The introduction of a once-weekly insulin formulation represents a significant shift in the paradigm of insulin therapy, offering a more flexible and patient-friendly treatment option.	90.9%
Place in therapy of once-weekly basal insulin analogs	
20. Once-weekly basal insulin should be considered as an add-on to other therapies, such as metformin, GLP-1 receptor agonists, and SGLT-2 inhibitors, for people living with type 2 diabetes who have not achieved glycemic targets with these therapies alone, either in mono therapy or in combination.	90.9%
21. From a clinical standpoint, weekly basal insulin could play a central role in the treatment of patients with suboptimal control on daily basal insulin, helping to improve blood glucose levels with fewer injections.	81.8%
22. While the efficacy of weekly basal insulin has been established in clinical trials, real-world data will be crucial in determining its true place in therapy, especially in diverse patient populations.	90.9%
23. Combining once-weekly basal insulin therapy with other weekly therapies further simplifies diabetes management.	100%
Elegibility in once-weekly basal insulin analog	
24. All people living with type 2 diabetes requiring basal insulin are potential candidates for once-weekly basal insulin therapy.	90.9%
25. Once-weekly basal insulin can benefit both subjects with type 2 diabetes patients already on daily basal insulin who seek to simplify their treatment regimen and insulin-naïve subjects requiring initiation of insulin therapy.	100%
26. People living with type 2 diabetes already on daily basal insulin and achieving adequate metabolic control should not be switched to once-weekly insulin therapy.	18.2%
27. People living with diabetes who struggle with insulin administration, such as elderly patients supported by caregivers or those with particularly irregular lifestyles, are the primary candidates of once-weekly basal insulin therapy.	63.6%
28. Once-weekly basal insulin should be offered primarily to individuals on daily basal insulin therapy alone rather than to those on basal-bolus or basal-plus regimens.	63.6%
29. The implementation of once-weekly basal insulin therapy requires careful patient selection and monitoring, ensuring optimal outcomes for those who will benefit most from this novel approach.	45.5%
Challenges for once-weekly basal insulin analogs	
30. To initiate therapy with once-weekly basal insulin, it is essential to raise awareness about the starting dose, weekly titration, and the process for transitioning from daily basal insulins.	81.8%
31. The use of different titration algorithms, compared to second-generation basal insulins (degludec and glargine U300), poses a challenge in the implementation of once-weekly basal insulin therapy.	36.4%
32. To support the adoption of once-weekly basal insulin and address potential concerns, the implementation of educational programs for people living with type 2 diabetes is essential.	90.9%
33. People living with diabetes who engage in intense physical activity should not be treated with once-weekly basal insulin.	0%
34. Once-weekly basal insulin therapy may pose a challenge in cases of hospitalization and acute illnesses.	9.1%
35. Management of hypoglycemia is more challenging with once-weekly basal insulin.	0%
Future perspectives and technology	
36. The use of Continuous Glucose Monitoring (CGM) can be helpful in optimizing once-weekly insulin therapy.	63.6%
37. The use of dedicated digital tools can support accurate titration and help achieving glycemic targets.	81.8%
38. Once-weekly basal insulin is expected to progressively and completely replace daily basal insulin therapy.	81.8%

The statements that were rated by the expert panel are listed. Percent (%) achieved consensus refers to the rated scores 4 (agree) and 5 (strongly agree) in the Likert scale

## Discussion

The aim of this consensus study, conducted using a Delphi-like methodology, was to highlight the benefits and challenges of once-weekly administration of basal insulin in the treatment of T2D and to examine its potential impact on disease management.

The consensus supports the adoption of once-weekly basal insulin for people with T2D, including those already on daily insulin with good metabolic control. Once-weekly basal insulin may offer significant advantages in terms of adherence, quality of life, and clinical inertia, while concerns regarding its use in specific clinical scenarios, such as physical activity, hospitalization, and hypoglycemia management, appeared largely unfounded. De facto, in a recent economic evaluation of once weekly insulin icodec, these cost items are irrelevant [25].

Icodec is an acylated insulin analogue, developed to retain an attenuated insulin receptor (IR) binding affinity, a strong but reversible binding to human serum albumin, and an extended time-action profile with slow clearance, offering patients an ultra-long-acting once-weekly insulin that may require a one-time loading dose due to the longer time needed to reach steady state [11]. Furthermore, icodec exposure has been shown to retain similar glucose-lowering effect independently of the subcutaneous injection site, namely the thigh, abdomen and upper arm [30].

Promising results were obtained from phase 2 trials assessing the efficacy in terms of glycemic control and safety of icodec (compared with glargine U100) in insulin-naïve or insulin-treated T2D patients [31–33]. Subsequently, the ONWARDS research program was conducted, aimed at investigating once-weekly insulin icodec in more than 4000 diabetes patients (insulin-naïve, T1D and T2D patients). In six phase 3 trials, icodec was non-inferior to available basal insulins (glargine U100 and degludec) in reducing HbA1c from baseline, along with a safe and well-tolerated profile across all six trials [34]. Moreover, compared to control once-daily insulin analogues, icodec showed superiority in insulin-naïve patients (ONWARDS 1, 3, and 5) and in patients previously treated with basal insulin (ONWARDS 2), demonstrating its superior clinical efficacy.

Importantly, while no significant difference in hypoglycamia rates was reported among T2D patients in any of the ONWARDS1-5 studies, data on T1D patients in the ONWARDS 6 study revealed that the rates of a clinically significant or severe hypoglycemia were significantly higher with insulin icodec compared with degludec [35]. Specifically, during weeks 22–26 of the study, the mean time below 3·0 mmol/l (< 54 mg/dl) was on the threshold of the internationally recommended target of < 1%, and below the target during weeks 48–52 of the study. Severe hypoglycemia was reported as follows: from baseline to week 26, 47 episodes in 9 of 290 participants receiving icodec and 17 episodes in 9 of 290 participants receiving degludec; while an evaluation over 57 weeks revealed 56 episodes in 13 participants receiving icodec and 25 episodes in 12 participants receiving degludec. Previous hypoglycemic unawareness was not reported. Nonetheless, icodec showed non-inferiority to once-daily degludec in Hb1Ac reduction at week 26. As for CGM, the recommended target of more than 70% Time In Range (TIR) was not met in either group. The authors concluded that further studies are needed to better understand the potential use of insulin icodec in the management of T1D patients.

A meta-analysis assessing the efficacy and safety of icodec in the ONWARDS 1–5 trials revealed that once-weekly insulin icodec administration yielded a greater HbA1c reduction [mean difference − 0.17%, 95% confidence interval (CI; -0.28 to -0.06), *p* = 0.003] and a higher proportion of patients who achieved HbA1c < 7% without hypoglycemia [odds ratio 1.45, 95% CI (1.26–1.67), *p* < 0.00001] compared with once-daily basal insulin analogues [36]. The meta-analysis also confirmed the absence of major safety concerns. Indeed, there was a significantly higher incidence of level 1 hypoglycemia (≥ 54 and < 70 mg/dl) but no significant difference for the incidence of level 2 (< 54 mg/dl), severe hypoglycemia or combined level 2 and severe hypoglycemia) with insulin icodec.

In a subsequent participant-level post-hoc meta-analysis of the ONWARDS 1–5 trials [37], the incidence of hypoglycemia was similar in the icodec and the comparators groups, icodec was associated with higher rates of clinically significant hypoglycemia equaling one additional episode every 6 years, and fewer severe hypoglycemic episodes, reassuring the overall data. Importantly, the efficacy of icodec was confirmed as it yielded a greater reduction in HbA1c (estimated treatment difference range [-0.10 to -0.29%]; all *p* < 0.05). The reduction of HbA1c was, though, associated with an increase in weight gain, a known side effect of insulin that may influence adherence to therapy [38].

Once-weekly insulin icodec has the potential to offer better glucose levels, prolonged exposure, and consistent levels of insulin, which reduces fluctuations in insulin concentration observed with daily basal insulins and improve glycemic variability [39]. In addition, a once-weekly regimen can be beneficial in case of a missed dose or accidental dose duplication [39].

Yet another meta-analysis of 7 trials assessing the efficacy of icodec compared with degludec and glargine in 3286 T2D patients was performed [40]. Data assessment allowed the authors to conclude that icodec does improve HbA1c (mean difference − 0.15%; 95% CI -0.21, -0.10; *p* < 0.0001) and TIR (TIR mean difference 2.83%; 95% CI 0.94; 4.71; *p* = 0.003) compared with once-daily insulin therapy. With regards to the TIR, the authors observed that there is a potential clinical advantage in mitigating glycemic variability and improving overall glucose management if CGM data is incorporated into the assessment of basal insulin efficacy and optimization of disease management.

The use of CGM has been shown to be associated with improvement of HbA1c, reduction of hypoglycemia and diabetes-related stress, as well as treatment satisfaction, and is predicted to, in combination with simplified regimens such as once-weekly insulin icodec, improve patient care and clinical outcomes in diabetes management [22]. Thus, the efficacy of icodec can be assessed using TIR and CGM data together, suggesting that the use of CGM can enhance therapeutic efficacy.

As for diabetes patients being hospitalized, a post-hoc analysis revealed that 127 patients enrolled in the ONWARDS 1–5 trials were hospitalized, for a total of 152 hospitalizations (77 medical and 75 surgical) [41]. The safety and efficacy of icodec was confirmed in the setting of hospitalized diabetes patients, as glycemic levels were stably maintained and treatment with icodec was not interrupted during hospitalization.

Another post-hoc analysis evaluating the occurrence of physical activity-related hypoglycemia in the ONWARDS 1–5 trials revealed that physical activity-related level 2 or 3 hypoglycemic episodes were low in all but the ONWARDS 4 trial [42]. No statistically significant differences were observed between icodec and comparators in the odds of experiencing level 2 or severe hypoglycemia related to physical activity.

Further assessments performed across all ONWARDS 1–5 trials confirmed that there is no additional risk of experiencing hypoglycemic episodes attributed to physical activity with once-weekly icodec compared with once-daily basal insulins in adult T2D patients [43].

The concomitant use of GLP-1RA in the ONWARDS 1–5 trials was assessed post-hoc and showed that the efficacy and safety of icodec were consistent, irrespective of baseline GLP-1RA use [44]. Larger or similar Hb1Ac reductions and low rates of clinically significant or severe hypoglycemic events were seen in the icodec group compared with comparators, and lower hypoglycemia rates among GLP-1RA users in ONWARDS 4.

Once-weekly basal insulin is predicted to facilitate and simplify the integration with once-weekly GLP-1RA. The phase 3 COMBINE clinical development program 1–4 is currently evaluating the efficacy and safety of the fixed dose combination between once-weekly icodec and once-weekly semagutide [11].

Assessment of patient-reported outcomes in terms of treatment satisfaction, well-being and quality of life are of great importance and reveal to which extent these clinical endpoints affect treatment adherence and persistence. In fact, higher insulin treatment satisfaction due to a lower treatment burden has been linked to increased adherence [45].

Treatment satisfaction scores from ONWARDS 2 and 5 have been evaluated and are reported to be higher among patients on inulin icodec regimen compared to patients in comparator groups [41]. Also, compliance scores were higher for patients receiving insulin icodec in the ONWARDS 5 trial.

Patient- and provider preferences in a scenario of a once-weekly basal insulin were recently evaluated in a survey-based study involving 401 patients (both insulin-naïve and insulin experienced) and 362 healthcare providers [46]. Remarkably, 91% of patients and 89% of healthcare providers would choose a once-weekly basal insulin product over another type of basal insulin.

Economic implications of once-weekly basal insulin therapy were another key focus of the panel discussion. Although statement 18 did not meet the consensus threshold (63.3%), real-world economic data support its cost-saving potential. A cited five-year budget impact analysis of the adoption of once-weekly insulin icodec for the Italian NHS projected cumulative savings of €37.9 million, derived from reduced needle use (€127 million) and improved adherence and administration efficiency, despite an initial increased expenditure of €113.9 million for icodec acquisition [26]. Icodec was recently approved by the Italian Medicines Agency (AIFA) for the treatment of adults affected by type 2 and type 1 diabetes [47].

Statement 14, which reached full consensus, highlighted a substantial reduction in injection burden (from 365 to 52 annually), improving adherence and treatment acceptance, with an estimated per-patient annual saving of €105.95 [25]. Furthermore, in statement 17 (90.9% agreement), once-weekly insulin was recognized for its potential to reduce healthcare staff workload, particularly in inpatient and long-term care settings, yielding an additional saving of up to €374 per patient annually. Sensitivity analyses confirmed that adherence was the primary economic driver, underscoring the robustness of the projected savings. Moreover, a real-world study demonstrated the cost-effectiveness of switching from other basal insulins, showing dominance of icodec over insulin degludec under routine care [48].

Although expert agreement on broader healthcare system savings was not unanimous, these findings indicate that the implementation of once-weekly basal insulin could offer meaningful economic value when considering both direct- and indirect cost offsets. Considering the global economic benefits generated by reduced needle use, improved adherence and cumulative savings on staff costs, once-weekly insulin icodec grants no incremental costs but potential savings [25].

Finally, although promising clinical trial results forecast that once-weekly insulin icodec will be well-received by patients and healthcare professionals based on its clinical outcomes, independently of patient subgroup, pathophysiology, ethnicity or race [49–51], further data from the real-world will be essential to fully establish icodec’s place in therapy and individualized care strategies. Regarding the importance of real-world data, an augmented study was conducted as a post-hoc analysis of the ONWARDS 5 trial aimed at evaluating the efficacy of icodec in a larger cohort of patients with real-word data from the US Ambulatory Electronic Medical Records (AMER) database, showed that the combination of clinical trial data with real-world matched data further underscored the efficacy of icodec in a real-world setting of diabetes management [52]. Specifically, additional studies are needed to understand the long-term efficacy, safety and tolerability among different patient demographics, range of comorbidities, in patients with changing insulin needs, or patients receiving various combination therapies. The uncertainty regarding T1D also necessitates further investigation. In addition, education of physicians, healthcare providers, caregivers and patients is crucial in the transition and implementation of once-weekly insulin regimens.

## Conclusion

Collectively, literature data support the use of once-weekly insulin icodec in the management of diabetes. Thanks to the reduced dosing frequency, this highly relevant therapeutic advancement is predicted to lower the burden on caregivers and the burden experienced by patients. Relieving patient- and clinician barriers, this new opportunity will likely improve adherence and persistence to therapy, thereby providing effective glycemic management, increase patient satisfaction and improve their quality of life [12, 20, 22, 41, 53]. Specifically, a reduction in treatment burden and improvement of adherence and quality of life has been forecasted upon weekly basal insulin treatment, as deduced from the outcomes of a survey aimed at inquiring the perspective of patients, physicians and payers [24]. The introduction of once weekly insulin icodec in the Italian clinical practice may then represent an economically sustainable and operationally advantageous strategy for the NHS [25]– [26].
